# Computational screening of walnut (Juglans regia) husk metabolites reveals Aesculin as a potential inhibitor of pectate lyase Pel3: Insights from molecular dynamics and τRAMD

**DOI:** 10.1016/j.bbrep.2025.102171

**Published:** 2025-07-23

**Authors:** Ali Khakpour, Negar Ahmadi Shadmehri, Amir Sedaghati, Hassan Jamshidian, Shamim Ghiabi, Payam Baziyar, Ehsan Heidari-Soureshjani, Seyedeh Atefeh Mirahmadi

**Affiliations:** a Department of Biology, Faculty of Science, University of Guilan, Rasht, Iran; b Department of Biology, Faculty of Science, University of Sistan and Baluchestan, Zahedan, Iran; c Blood Transfusion Research Center, High Institute for Research and Education in Transfusion Medicine, Tehran, Iran; d Isfahan Blood Transfuion Center, Isfahan, Iran; e Department of Medical Chemistry, Faculty of Pharmacy, Tehran Medical Sciences, Islamic Azad University, Tehran, Iran; f Department of Molecular and Cell Biology, Faculty of Basic Sciences, University of Mazandaran, Babolsar, Iran; g Department of Biology, Faculty of Science, Shahrekord University, P. O. Box. 115, Shahrekord, Iran; h Central Laboratory, Shahrekord University, Shahrekord, Iran; i Department of Fisheries, Faculty of Environment and Fisheries, University of Tehran, Iran

**Keywords:** Pectate lyase (Pel3), Aesculin, Molecular docking, MD simulation, Food spoilage

## Abstract

Pectate lyase (Pel3), an enzyme derived from *Clostridium* bacteria, plays a significant role in the degradation of pectin and contributes to the spoilage of agricultural products. Pel3 can bind to pectin and break it down, a process that accelerates food decay. Aesculin, a natural compound extracted from walnut husk, has been recognized for its antibacterial and antifungal properties, making it a promising natural inhibitor. The aim of this study was to investigate the inhibitory mechanisms of Aesculin through molecular simulations and random accelerated molecular dynamics (RAMD). Molecular docking results showed that Aesculin may effectively bind to Pel3 and form a strong interaction. RMSD analysis revealed that Aesculin's binding to Pel3 reduced structural fluctuations, thereby enhancing the enzyme's structural stability. Slight changes in the radius of gyration (Rg) indicate a decrease in structural compactness in specific regions of the protein. Furthermore, SASA analysis revealed a modest increase in solvent accessibility. RAMD simulations, performed with 120 replicates, showed a short average residence time (∼0.015 ns), suggesting rapid unbinding and weak interaction at the active site. MM-PBSA analysis yielded a total binding free energy of −2.92 ± 0.44 kcal/mol, mainly driven by van der Waals and electrostatic contributions, confirming moderate and reversible binding. These findings suggest that Aesculin may form alternating interactions with Pel3 as an effective natural inhibitor and exhibit a short residence time in its active site. The molecular dynamics simulations and RAMD analysis suggest that Aesculin can enhance the structural stability of Pel3, presenting it as a potential anti-spoilage agent in the food and agricultural industries.

## Highlights

• Pectate lyase (Pel3), an enzyme derived from Clostridium bacteria, plays a significant role in the degradation of pectin.
• Aesculin, a natural compound extracted from walnut husk, has been recognized for its antibacterial and antifungal properties.
• Aesculin forms alternating interactions with Pel3 and could be presented as a potential inhibitor in the food and agricultural industries.

## 1 Introduction

Pectate lyase (Pel) is an important enzyme produced by various phytopathogenic bacteria and fungi, including *Pectobacterium atrosepticum*, *Pectobacterium carotovorum*, and *Colletotrichum gloeosporioides*. It catalyzes the cleavage of pectin, a major component of plant cell walls, through a β-elimination mechanism, leading to tissue maceration and disease development, such as potato soft rot and avocado fruit rot. *P. carotovorum*, a Gram-negative, facultatively anaerobic bacterium, degrades plant cell walls through pectolytic enzymes like Pel, contributing to significant post-harvest losses. Inhibiting Pel activity is a promising strategy to reduce the virulence of these pathogens and manage related plant diseases [1,2].

Pel3, a pectate lyase, exhibits 67 % sequence identity with PelI but is not recognized by the Type II Secretion System (T2SS) of Dickeya dadantii. T2SS is a highly specialized protein export mechanism in Gram-negative bacteria, responsible for translocating folded enzymes and virulence factors across the outer membrane. The secretion process of Pel3 begins with its translocation across the inner membrane via either the Sec or Tat system, with Sec accommodating unfolded proteins and Tat transporting fully folded ones. Once in the periplasm, Pel3 engages with key T2SS components, including GspC and GspD, which mediate its passage through the outer membrane. Despite sharing a conserved overall topology with PelI—comprising an N-terminal fibronectin type III (Fn3) domain and a catalytic β-helix domain—Pel3 exhibits distinct structural variations in exposed loop regions, particularly loop 3 of Fn3. These structural features serve as secretion determinants, dictating T2SS recognition and ultimately governing the efficient export of Pel3 [3,4]. The full-length Pel3 was crystallized into two monoclinic forms, Pel31m and Pel32m, each containing one or two monomers in the asymmetric unit. Their structures were resolved at 1.8 and 2.1 Å resolution, respectively. Both structures share a compact, pear-shaped architecture, consisting of two distinct domains: a small fibronectin type III (Fn3) domain and a larger catalytic domain with a β-helix fold (residues 120–347). These domains are connected by a decapeptide segment (residues 110–119), which maintains a consistent orientation in both crystal forms [5]. The catalytic domain of Pel3 exhibits the characteristic β-helix fold of the PL-3 family, made up of eight right-handed coils stabilized by a hydrophobic core, formed by Ile, Val, and Leu residues arranged in regular ladders. Five conserved disulfide bonds reinforce the β-solenoid fold, stabilizing extended loops. The catalytic site features invariant residues Lys227, Lys252, and Arg255, essential for substrate binding and catalysis. A sulfate ion observed in Pel31m mimics the natural substrate, polygalacturonic acid, by interacting with these residues. Additionally, a structural calcium ion in monomer B of Pel32m stabilizes loop T3.4, a unique arrangement to Pel3 and PelI that is absent in other PL-3 members [6]. The Fn3 domain of Pel3, a seven-stranded fold, is structurally similar to that of PelI, with slight deviations. It consists of two antiparallel β-sheets. Unlike most PL-3 family members that contain carbohydrate-binding or lectin-like domains, Pel3 and its close homologs feature this Fn3 domain, whose biological function is not entirely understood. It likely contributes to plant cell wall degradation or secretion via the type II secretion system (T2SS). Structural analysis suggests that specific loops in the Fn3 domain, particularly loops 3 (residues 42–51) and 5 (residues 85–94), are essential for secretion. These loops form a continuous interface, potentially acting as a secretion signal that guides Pel3's recognition and transport by the T2SS [7]. Natural compounds have gained significant attention as potential inhibitors of Pel, offering an environmentally friendly alternative to chemical control methods. Among the natural inhibitors, oleuropein, a phenolic compound found in olive leaves, has shown significant antibacterial activity against *P. atrosepticum* with an IC50 of 0.2 mg/mL. This suggests that Oleuropein could be a potent candidate for bacterial growth inhibition and might help in managing soft rot caused by *P. atrosepticum*. Similarly, extracts from Ceratonia siliqua (locust tree) leaves have demonstrated the ability to inhibit pectate lyase activity and reduce the virulence of *P. atrosepticum*, suggesting their potential use in controlling potato soft rot [8]. Epicatechin, a flavan-3-ol present in unripe avocado fruit, has also been identified as a natural inhibitor of pectate lyase activity. With a Ki of 3.4 μM, epicatechin effectively reduces the macerating ability of PL from *C. gloeosporioides*. At higher concentrations, which are naturally present in unripe avocado fruit, epicatechin significantly inhibits Pel activity, making it a potential factor contributing to the resistance of unripe avocado fruits to fungal pathogens. The inhibition of Pel, particularly by natural compounds, is an important strategy for mitigating the effects of plant diseases caused by pectinolytic organisms. These natural inhibitors not only offer a sustainable approach to disease control but also reduce the need for synthetic chemicals, making them an attractive option for integrated pest management systems. Additionally, further exploration of such compounds could lead to the development of novel plant protection agents with reduced environmental impact [9]. The green husks of Juglans regia (walnut) are rich in bioactive compounds, making them a valuable source of natural antioxidants, antimicrobials, and potential pharmaceutical agents. Phytochemical analysis has identified a wide range of secondary metabolites, including flavonoids, phenolic acids, and glycosides. In a 2023 review article, Ahmad Bhat and et al., identified Aesculin, Taxifolin, Pantocid, Quercetin, Glucronide, Kaempferol, Rhamnoside, Syringetin-O-Hexoside, Myricetin-3-O-glucoside, Myricetin-3-O-pentoside, and Epicatechin as the most important compounds found in Juglans regia (walnut). Natural compounds play a crucial role in pharmaceutical and nutritional applications due to their antioxidant [10], anti-diabetic [11], anti-amyloidogenic [[12], [13], [14], [15], [16]], anti-cancer [17], anti-Alzheimer's [18], anti-inflammatory [19], protective properties against oxidative stress [20], and Therapeutic effect on metabolic diseases [21]. Since their paper is a systematic review of previous studies, its significance lies in consolidating and analyzing existing data to emphasize the biological effects of these compounds [22]. Among these bioactive molecules, Aesculin, a coumarin glucoside, exhibits antioxidant and anti-inflammatory properties. Taxifolin and Quercetin, two flavonoids, are known for their strong free radical-scavenging activities, while Kaempferol and its glycosylated derivatives (Rhamnoside and Syringetin-O-Hexoside) contribute to anti-inflammatory and antimicrobial effects. Myricetin-3-O-glucoside and Myricetin-3-O-pentoside have been recognized for their antioxidative and potential neuroprotective roles. Additionally, Pantocid (Halazone) demonstrates antimicrobial activity, and Epicatechin, a well-known flavanol, serves as a reference compound for antioxidant studies. These findings highlight the potential of walnut green husks as a rich source of bioactive molecules for pharmaceutical and agricultural applications [22]. τ-Random Acceleration Molecular Dynamics (τRAMD) is a powerful computational method for estimating the relative residence times of protein-ligand complexes. By applying a randomly oriented force to the ligand's center of mass, this approach enhances the dissociation process without requiring prior knowledge of the exit pathway, enabling the efficient exploration of unbinding events on the nanosecond timescale. Beyond characterizing dissociation mechanisms, τRAMD provides insights into ligand stability and its retention within the protein-binding site. Extensively validated across diverse protein systems, including soluble and membrane-associated proteins, this method has demonstrated strong agreement with experimental data, highlighting its reliability and broad applicability in studying ligand-protein interactions [23,24]. This study aims to computationally investigate the inhibition and dissociation mechanisms of Pectate Lyase Pel3 by bioactive compounds derived from Juglans regia L. green husks, including Aesculin, Taxifolin, Pantocid, Quercetin, Glucronide, Kaempferol, Rhamnoside, Syringetin-O-Hexoside, Myricetin-3-O-glucoside, Myricetin-3-O-pentoside, and Epicatechin, using molecular docking studies. The compounds with the best binding energies in docking studies will then be subjected to τRAMD simulations to further explore their dissociation mechanisms and potential as effective Pel3 inhibitors for controlling plant diseases caused by pectinolytic bacteria.

## 2 Methods

### 2.1 System preparation

Based on the comprehensive phytochemical review by Ahmad Bhat et al. (2023) [22], which highlighted the presence of several bioactive compounds in Juglans regia (walnut) husk with antioxidant, anti-inflammatory, and oxidative stress-mitigating properties, a panel of key constituents was selected for molecular docking analysis. The selected compounds were chosen for their reported biological relevance and relative abundance in walnut green husks, with a specific focus on their potential to act as natural inhibitors of the Pel3 enzyme, a virulence factor implicated in food spoilage. The three-dimensional (3D) structures of eleven bioactive compounds were retrieved from the PubChem database (https://pubchem.ncbi.nlm.nih.gov) in SDF format. These included Aesculin (CID: 5281417), Taxifolin (CID: 439533), Pantocid (Halazone) (CID: 3552), Quercetin (CID: 5280343), Glucronide (CID: 5319484), Kaempferol (CID: 5280863), Rhamnoside (CID: 14299117), Syringetin-O-Hexoside (CID: 14524434), Myricetin-3-O-glucoside (CID: 5318606), Myricetin-3-O-pentoside (CID: 21477996), and Epicatechin (CID: 72276). These compounds were energy-minimized and prepared for docking using standard protocols to ensure proper geometry and protonation states at physiological pH. The Pel3 enzyme structure was prepared by removing crystallographic water molecules and adding hydrogen atoms, followed by optimization to ensure compatibility with docking simulations. All molecular structures were optimized using Chimera 1.18, applying Gasteiger charges and the AMBERff14SB force field. The 3D structure of Pel3 was obtained from the RCSB Protein Data Bank (PDB ID: 4U4B) and processed in PDB format. Non-relevant water molecules and external ligands were removed, and missing loops and side chains were refined using Modeller 10.3. Protonation states were assigned at pH 7.5 using PDB2PQR, corresponding to physiological conditions.

### 2.2 Molecular docking

Molecular docking simulations were performed using AutoDock 4.2 (Morris et al., 2009) to assess the binding affinity of these compounds to Pel3. The docking grid was defined with a 120 × 120 × 120 Å^3^ grid box centered at the Pel3 active site, ensuring full coverage of the binding pocket. A total of 200 runs and 10 × 10^6^ evaluations were performed using the Lamarckian Genetic Algorithm to determine the most favorable binding conformations. To enhance docking accuracy, additional docking simulations were conducted using AutoDock Vina 1.2 [25]. The binding modes and docking scores were analyzed, and the compounds with the highest binding affinity were selected as candidates for τRAMD simulations to further investigate their dissociation mechanisms and inhibition potential against Pel3 [25].

### 2.3 Molecular dynamics simulations

Each protein-ligand complex was subjected to three independent 100 ns unrestrained Molecular Dynamics (MD) simulations, accumulating a total simulation time of 3.9 μs. The CHARMM36 Force Field was used for all simulations, ensuring accurate representation of molecular interactions. Ligand topologies were generated using CHARMM-GUI (https://www.charmm-gui.org), where atomic partial charges were automatically assigned. The simulation systems were solvated in a periodic cubic box with TIP3P water molecules, maintaining a minimum buffer of 1.0 nm between the protein and the box boundaries. Sodium and chloride ions were added to achieve charge neutrality and an ionic concentration of 0.15 M. Among the docked compounds, Aesculin exhibited the most favorable binding free energy and was therefore selected for subsequent MD and Random Accelerated Molecular Dynamics (RAMD) simulations to further characterize its stability and unbinding kinetics within the Pel3 binding pocket. To equilibrate the systems, an initial energy minimization step was performed, followed by equilibration in both canonical (NVT) and isothermal-isobaric (NPT) ensembles, as previously reported [26]. The temperature was set to 300 K using the V-rescale thermostat, while the pressure was maintained at 1.0 bar through the Parrinello-Rahman barostat. The LINCS algorithm was employed to constrain hydrogen-involving bonds, ensuring numerical stability. Long-range electrostatic interactions were computed using the Particle Mesh Ewald (PME) method, with a uniform cut-off of 1.2 nm applied to both electrostatic and Lennard-Jones interactions, as previously reported [[27], [28], [29]]. Post-simulation analyses were conducted using GROMACS 2020.1 built-in tools, while hydrophobic interaction mapping was performed with the Protein-Ligand Interaction Profiler (PLIP). Structural visualizations and graphical plots were generated using PyMOL v2.5.4 and Gnuplot v5.2, respectively.

### 2.4 Binding free energy calculation using MM-PBSA

To quantitatively estimate the binding free energy of the Pel3–Aesculin complex, the Molecular Mechanics Poisson–Boltzmann Surface Area (MM-PBSA) method was employed using gmx_MMPBSA v1.5.0.3, based on the AmberTools MMPBSA.py v16.0 framework. Snapshots were extracted from the equilibrated portion of the 300 ns molecular dynamics trajectory at 100 ps intervals, providing a representative ensemble for energy analysis. The calculations included van der Waals, electrostatic, polar solvation, and non-polar solvation energy components. The non-polar solvation term was estimated using the solvent-accessible surface area (SASA) model. All computations were performed using GROMACS topology and trajectory files to ensure consistency with the simulation environment. This approach offers a thermodynamically relevant estimation of ligand–receptor interaction strength in an implicit solvent model.

### 2.5 τ-Random Acceleration Molecular Dynamics (τRAMD)

The final configurations from the three independent 100 ns MD simulations served as initial structures for τ-Random Acceleration Molecular Dynamics (τRAMD) simulations. For each system, 120 independent τRAMD dissociation trajectories were generated per replica, culminating in a total of 720 τRAMD simulations. A predefined force of 879 kJ mol^−1^ nm^−1^ and 586 kJ mol^−1^ nm^−1^ was applied to the center of mass (LIGcom) of nucleoside and non-nucleoside inhibitors, respectively. The force direction was reassigned stochastically if LIGcom displacement remained below 0.0025 nm over 50 integration steps (100 fs). Dissociation trajectories were terminated once the ligand center of mass reached a separation of 5.0 nm from the binding site. To determine relative residence times (τ), time-distribution analyses were performed on the τRAMD trajectories. Logarithmic values of both computed and experimental residence times were normalized using a min-max scaling approach. The τRAMD protocol was implemented in GROMACS, utilizing the RAMD-GROMACS module, which is fully compatible with the CHARMM36 Force Field. Ligand dissociation pathways were analyzed using distance-based clustering, and critical binding site interactions were identified based on geometric and energetic parameters. To enhance statistical robustness, residence time estimations were further validated via bootstrapping, ensuring reliability and reproducibility of the findings [30]. Aesculin is a coumarin that has been found in A. hippocastanum and has antioxidant and anti-inflammatory activities.1,2 It reduces the size of gastric ulcers and decreases increases in the levels of malondialdehyde (MDA) and the activity of myeloperoxidase (MPO) and catalase in a mouse model of ethanol-induced ulcers when used at a dose of 25 mg/kg.1 Esculin (20 mg/kg) decreases LPS-induced increases in the levels of TLR4, MyD88, IRAK4, and phosphorylated NF-ĸB in the lung, as well as levels of IL-1β, IL-6, and TNF-α in the bronchoalveolar lavage fluid (BALF) in a mouse model of acute lung injury [31,32].

## 3 Result and dissection

### 3.1 Binding mode and interaction analysis of Aesculin with Pel3 enzyme

To evaluate the binding affinity and interaction profile of the Aesculin molecule with the Pel3 enzyme, molecular docking simulations were performed. The docking results revealed a moderately stable interaction between the ligand and the protein, characterized by a calculated binding free energy (ΔG_bind) of −6.39 kcal/mol, corresponding to an estimated inhibition constant (Ki) of 20.56 μM at 298.15 K. These values suggest a medium-strength and potentially reversible interaction. When compared to other phytochemical constituents of Juglans regia extract, Aesculin demonstrates a comparable binding affinity. For example, compounds such as Epicatechin (−6.29 kcal/mol, Ki = 8.84 μM), Glucuronide (−6.23 kcal/mol, Ki = 3.59 μM), and Quercetin (−6.26 kcal/mol, Ki = 11.13 μM) show slightly lower or similar ΔG_bind values, with some exhibiting stronger theoretical inhibitory potential (i.e., lower Ki values). Notably, Glucuronide and Myricetin glycosides exhibit more favorable intermolecular and van der Waals interaction energies, suggesting they may interact more effectively with the protein's binding pocket. Furthermore, Aesculin's intermolecular energy (−8.78 kcal/mol) and van der Waals plus hydrogen bond and desolvation energy (−8.38 kcal/mol) indicate that non-covalent interactions, especially hydrogen bonding, play a key role in ligand stabilization. The electrostatic component (−0.40 kcal/mol) is minimal, suggesting limited contribution from charged interactions. The torsional free energy (2.39 kcal/mol) reflects the ligand's conformational flexibility upon binding. While Aesculin ranks among the top candidates in terms of ΔG_bind, compounds such as glucuronide and Myricetin-3-O-pentoside display slightly more favorable Ki values (3.59 μM and 4.96 μM, respectively), warranting further comparative studies and dynamic simulations.

**Fig. 1:**
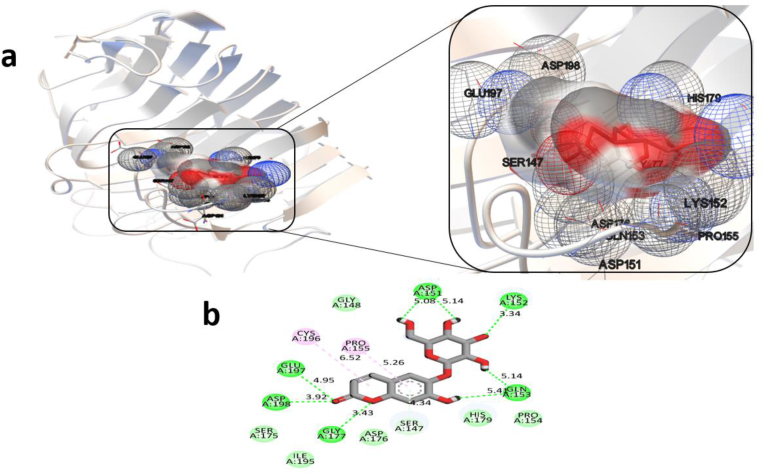
Three-dimensional interaction diagram (a) and two-dimensional structural orientation (b) of the Aesculin–Pel3 enzyme complex.

Overall, these results position Aesculin as a moderately potent ligand with stable interaction characteristics, although other compounds in the extract may exhibit stronger predicted inhibitory effects. The detailed energy parameters obtained from the docking simulations for all compounds are summarized in Table 1. The interaction map between Aesculin and Pel3 (Fig. 1a) provides key insights into the molecular contacts that stabilize the complex. Aesculin forms several conventional hydrogen bonds with polar amino acid residues located in the active site cleft of Pel3, which likely contribute significantly to the observed binding affinity. Notably, Asp151, Lys152, and Gln153 establish multiple hydrogen bonds at distances ranging from 3.34 to 5.41 Å, which help anchor the sugar moiety of the ligand. The hydroxyl and carbonyl groups of Aesculin serve as hydrogen bond acceptors in these interactions. Asp198 and Glu197, located deeper within the binding site, also engage in hydrogen bonding interactions (3.92 and 4.95 Å respectively), likely stabilizing the aromatic core of Aesculin and contributing to its orientation. These polar interactions are complemented by π-alkyl and van der Waals contacts, which enhance the overall stability of the complex through hydrophobic contributions. For instance, Pro154, Pro155, and Cys196 participate in π-alkyl interactions with the aromatic system of Aesculin at relatively long-range distances (5.26–6.52 Å), which may still play a stabilizing role due to the flexibility of these residues and partial stacking effects. Furthermore, Ser147 appears to participate in a π-donor hydrogen bond, a weaker but structurally relevant contact that may assist in further orienting Aesculin's aromatic rings. Several additional residues—including Asp176, Gly177, Gly148, Ser175, Ile195, and His179—form non-specific van der Waals interactions, which help shape the hydrophobic pocket surrounding the ligand and may contribute to entropic stabilization (Fig. 1b). From an energetic perspective, the intermolecular energy was calculated at −8.78 kcal/mol, primarily driven by van der Waals and hydrogen bonding interactions (−8.38 kcal/mol), with minimal contribution from electrostatics (−0.4 kcal/mol). The torsional free energy of +2.39 kcal/mol represents the entropic cost associated with conformational restrictions of Aesculin upon binding. The internal energy of the ligand in its bound state was found to be −3.71 kcal/mol, which is equal to the unbound system energy, implying that strain within the ligand itself does not significantly affect binding free energy (see Table 1 for numerical details). Taken together, these observations suggest that Aesculin engages with the Pel3 enzyme through a combination of polar and hydrophobic interactions, with a relatively short residence time and moderate binding energy. Although several stabilizing interactions are evident, the overall affinity is limited, possibly due to suboptimal shape complementarity or dynamic behavior of the binding site. The results of our previous study highlighted the promising antibacterial potential of various plant-derived extracts against phytopathogenic bacteria responsible for significant agricultural losses. Among the tested materials, extracts from Juglans regia (husk) and Vicia faba (outer peel) exhibited broad-spectrum antibacterial activity, effectively inhibiting the growth of all four studied bacterial strains: Pectobacterium carotovorum, Dickeya chrysanthemi, Pseudomonas syringae, and Ralstonia solanacearum. In contrast, Citrus sinensis (peel) and Urtica urens extracts displayed selective inhibitory effects, primarily targeting P. syringae and R. solanacearum. The inhibition zones observed (8–14 mm) and the MIC/MBC values (6.25 and 12.5 mg/mL, respectively) suggest that these natural extracts contain bioactive compounds with potential utility as eco-friendly antibacterial agents. These findings are consistent with previously reported antimicrobial properties of agricultural waste products and emphasize their applicability in developing sustainable biocontrol strategies. Particularly, the broad efficacy of J. regia husk extract supports its selection for further mechanistic and computational studies, such as molecular docking and dynamics simulations, to better understand its inhibitory interaction with plant-pathogenic enzymes like Pel3. Continued exploration of such natural compounds could pave the way for the development of environmentally safe alternatives to synthetic pesticides in agricultural systems [33].

**Table 1:** Docking results of selected compounds from Juglans regia hull extract with the Pel3 enzyme, including binding energy and key interaction parameters.

Compounds	Estimated Binding Free Energy (ΔGbind)	Inhibition Constant (Ki)	Intermolecular Energy	vdW + H-bond + desolvation Energy	Electrostatic Energy	Internal Energy of Complex	Torsional Free Energy	Energy of Unbound System
Aesculin	−6.39	20.56	−8.78	−8.38	−0.4	−3.71	2.39	−3.71
Epicatechin	−6.29	8.84	−8.68	−8.58	−0.11	−1.66	1.79	−1.66
Glucronide	−6.23	3.59	−10.41	−9.37	−1.04	−3.34	2.98	−3.34
Kaempferol	−6.12	10.01	−8.31	−8.2	−0.11	−1.66	1.49	−1.65
myricetin 3-O-beta-d-glucopyranoside	−6.04	11.56	−10.61	−10.51	−0.1	−4.94	3.88	−4.94
Myricetin-3-O-pentoside	−6.22	4.96	−10.52	−9.63	−0.89	−4.28	3.28	−4.28
Pantocid	−6.16	30.36	−7.36	−7.4	0.04	−0.58	1.19	−0.58
Quercetin	−6.24	11.13	−8.55	−8.39	−0.15	−2.51	1.79	−2.51
Rhamnoside	−4.92	249.56	−11.18	−10.7	−0.48	−9.25	6.26	−9.25
Syringetin-3-O-hexoside	−6.27	21.27	−10.25	−10.14	−0.11	−5.03	3.88	−5.03
Taxifolin	−6.26	13.07	−8.45	−7.87	−0.58	−2.5	1.79	−2.5

### 3.2 Structural stability assessment via RMSD, Rg, and SASA

**Fig. 2:**
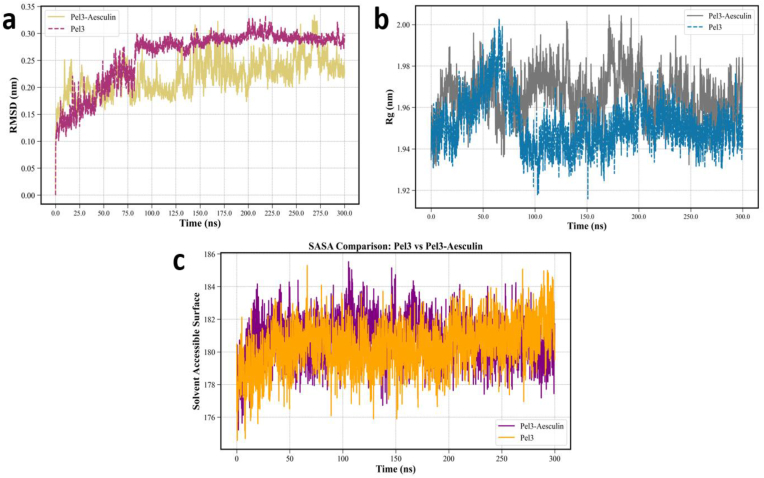
Structural stability assessment of Pel3 and the Pel3–Aesculin complex. (a) Root means square deviation (RMSD) profiles over the simulation time indicate overall structural stability, with the complex showing slightly lower fluctuations compared to the free protein. (b) Radius of gyration (Rg) analysis demonstrates a modest increase in compactness for the free protein, while the complex shows a slightly expanded conformation, possibly due to ligand accommodation. (c) Solvent-accessible surface area (SASA) analysis reveals comparable overall exposure, with local variations near the binding site suggesting conformational adjustments upon ligand binding.

MD simulations were conducted for 300 ns on both the *Pel3* enzyme and the *Pel3–Aesculin* complex to investigate structural fluctuations and stability changes at the atomic level. The system behavior was evaluated using three key structural descriptors: RMSD, Rg, and SASA. These parameters respectively reflect the structural stability, molecular compactness, and surface exposure of the protein to the aqueous solvent. A detailed understanding of these metrics provides critical insight into how ligand binding—in this case, *Aesculin*—influences the structural dynamics of the target protein. According to the RMSD plot in Fig. 2a, the *Pel3* protein exhibited more pronounced structural fluctuations during the early phase of the simulation (up to ∼50 ns) compared to the *Pel3–Aesculin* complex. Notably, fluctuations between 0 and 25 ns were especially significant in the unbound *Pel3*, indicating initial conformational adaptation in the absence of a ligand. These fluctuations gradually diminished, and the system reached a relatively stable state around 75 ns. In contrast, the complexed system displayed less pronounced initial deviations and exhibited a more stable behavior throughout the entire simulation. These observations are consistent with the numerical values shown in the accompanying table: the average RMSD for the unbound Pel3 was 0.26 ± 0.05 nm, while the *Pel3–Aesculin* complex exhibited a reduced RMSD of 0.22 ± 0.03 nm (Fig. 2a). Although the difference is subtle, it indicates enhanced structural stability of the protein upon ligand binding. Temporally, both systems reach a more uniform RMSD level after ∼75 ns; however, the Pel3–Aesculin complex consistently shows reduced fluctuation amplitude over time. In contrast, the unbound *Pel3* displays larger local fluctuations, reflecting greater conformational flexibility in the absence of the ligand. This suggests that ligand binding restricts structural dynamics and promotes conformational coherence. It can thus be inferred that ligand binding—either at the active site or other interaction interfaces—helps stabilize the protein's overall structure and dampens excessive motion. The Rg, which measures the distribution of atomic mass relative to the center of mass (Fig. 2b), supports these findings. Both systems displayed Rg fluctuations between 1.92 and 2.00 nm; however, the unbound Pel3 occasionally experienced sudden decreases in Rg, potentially indicative of local collapses or secondary structure rearrangements. In contrast, the Pel3–Aesculin complex showed a smoother Rg trajectory, remaining close to an average value of 1.97 nm for most of the simulation duration.

Quantitative data from Table 2 corroborate these results: the mean Rg for unbound *Pel3* was 1.95 ± 0.01 nm, while the complex had a slightly higher value of 1.97 ± 0.01 nm. Although this difference appears minimal, the increase in Rg for the complex may reflect a more open but stable conformation, possibly due to domain rearrangements upon ligand interaction. Furthermore, the Rg increase might arise from structural expansion in specific protein regions in response to Aesculin binding, leading to a greater distribution of atomic mass. Overall, the Rg analysis suggests that complex formation promotes greater structural stability and dynamic uniformity, potentially reducing the prevalence of highly flexible, extended conformations seen in the unbound protein. The reduced fluctuations in Rg imply fewer large-scale conformational transitions over time, a key indicator of system stability in biomolecular simulations involving ligand–protein interactions. The third parameter, SASA, provides insights into the extent of protein surface exposure to the solvent and is especially relevant for assessing the structural consequences of ligand binding. Changes in SASA may reflect conformational rearrangements or the opening/closing of surface-accessible sites. As shown in Fig. 2c, the average SASA of the complexed system was slightly lower than that of the unbound protein, although both systems exhibited fluctuations over time. According to the tabulated data, the mean SASA was 180.34 ± 1.43 nm^2^ for unbound *Pel3* and 180.77 ± 1.3 nm^2^ for the *Pel3–Aesculin* complex (Fig. 2c). While the numerical difference appears small, a closer look at the plots reveals that the complexed system had fewer abrupt fluctuations and a more stable average SASA. The unbound Pel3 showed occasional sharp decreases in SASA, possibly due to transient local collapses or the formation of looped or compact structures. These sharp declines were much less frequent in the presence of *Aesculin*, suggesting that ligand binding confers surface stability and helps preserve solvent exposure. As such, Aesculin binding may mitigate sudden structural contractions and support a more robust surface architecture—an important feature for protein functionality under physiological conditions. Structurally, the observed stability or mild increase in SASA upon ligand binding could indicate reorganization of surface domains, subtle spatial stretching, or even specific conformational openings induced by *Aesculin*. This aspect is particularly relevant to drug design, as stable complexes with consistent solvent-accessible surfaces are often better suited for molecular interactions, intracellular stability, and bioavailability.

**Table 2:** Comparative average values of structural stability parameters for the free Pel3 protein and the Pel3-Aesculin complex over 300 ns of MD simulation.

Protein/Complex	RMSD	Rg	RMSF	SASA	H-Bonds Protein-Solvation	H-Bonds Protein-Protein
Pel3	0.26 ± 0.05	1.95 ± 0.01	0.11 ± 0.09	180.34 ± 1.43	698.27 ± 17.81	251.57 ± 8.04
Pel3-Aesculin	0.22 ± 0.03	1.97 ± 0.01	0.11 ± 0.1	180.77 ± 1.33	695.76 ± 16.48	248.89 ± 7.52

### 3.3 Analysis of hydrogen bond in the Pel3 and Pel3–Aesculin systems

**Fig. 3:**
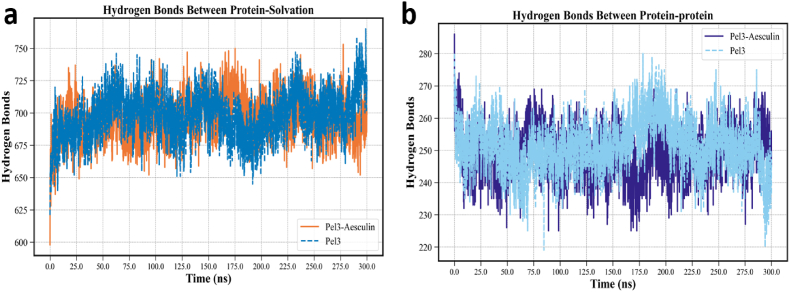
Hydrogen bonding analysis of the Pel3 protein and the Pel3–Aesculin complex. (A) Time evolution of hydrogen bonds between the protein and solvent molecules, showing overall similar hydration patterns in both systems, with minor fluctuations indicative of dynamic surface interactions. (B) Intramolecular hydrogen bonds within the protein structure, revealing increased internal stability in the complex compared to the free protein, likely due to ligand-induced structural tightening.

The study of hydrogen bond dynamics within biomolecular systems represents a fundamental approach to understanding intra- and intermolecular interactions during MD simulations. Hydrogen bonds, as one of the principal determinants of structural stability and spatial behavior in proteins, have direct implications on molecular mobility, solubility, secondary structure rearrangements, and overall system stability. In this context, two categories of hydrogen bonds were analyzed: (1) protein–solvent hydrogen bonds (Fig. 3a), and (2) intramolecular hydrogen bonds within the protein (i.e., between internal amino acid residues) (Fig. 3b). These two indices, in combination with RMSD, Rg, and SASA analyses, provide a comprehensive perspective on the structural stability of the Pel3–Aesculin complex relative to the unbound Pel3 protein.

Fig. 3A presents the variation in the number of hydrogen bonds between the protein and surrounding solvent over a 300 ns simulation for both the free Pel3 and the Pel3–Aesculin complex. As shown, both systems exhibit discernible fluctuations in the number of hydrogen bonds formed with the solvent throughout the simulation. However, the average values and the amplitude of these fluctuations differ between the two systems. According to the data in Table 2, the average number of proteins–solvent hydrogen bonds for the free Pel3 system is reported as 698.27 ± 17.81, whereas the corresponding value for the Pel3–Aesculin complex is slightly lower at 695.76 ± 16.48. Although the numerical difference appears modest, it indicates a meaningful alteration in solvent exposure upon ligand binding. The graph in Fig. 3A illustrates that, throughout most of the simulation time, the unbound Pel3 protein forms more hydrogen bonds with the solvent. This can likely be attributed to the greater solvent-accessible surface area of the unbound protein, which enables more extensive interactions with water molecules. In contrast, in the presence of Aesculin, certain surface regions of the protein may be engaged in ligand binding, thereby reducing their availability for hydrogen bonding with the solvent. This observation is consistent with the SASA data, where the Pel3–Aesculin complex demonstrated slightly higher average surface area values but reduced fluctuation amplitude, indicative of improved surface stability. Fluctuations in protein–solvent hydrogen bonding are particularly pronounced in the Pel3 system during specific simulation intervals—especially between 100 and 200 ns—where sharp declines and subsequent recoveries are observed. These abrupt changes may reflect localized structural rearrangements and the formation or disruption of domain-like structural motifs. Conversely, the hydrogen bonding profile of the Pel3–Aesculin complex displays a smoother trend with more gradual transitions, suggesting a more stable structural configuration in the aqueous environment. This smoother behavior likely results from reduced structural fluctuations in the presence of the ligand, supporting the idea of enhanced complex stability.

Further insight is gained from the analysis of Fig. 3b, which illustrates the number of intramolecular hydrogen bonds (i.e., within the protein structure). This measure provides a more direct assessment of how ligand binding affects the internal stability of the protein. According to the data, the Pel3–Aesculin complex consistently maintains a slightly higher number of interprotein hydrogen bonds compared to the free Pel3 protein. Numerical values from the table support this observation, with the average number of hydrogen bonds being 251.57 ± 8.04 for the unbound protein and 248.89 ± 7.5 for the complex. While the statistical difference is minor, the smoother trend and reduced fluctuations in the complex suggest a more stable hydrogen bonding network. The stability of internal hydrogen bonds is critically linked to the preservation of secondary and tertiary protein structure. A lower degree of fluctuation and the maintenance of a more constant number of hydrogen bonds throughout the simulation are indicative of improved structural cohesion in the ligand-bound form. In contrast, the free Pel3 system displays more pronounced variations in hydrogen bond counts, with recurring drops during the simulation, which could signify the disruption of α-helices, β-sheets, or other structural elements. These findings are also corroborated by other structural parameters. For instance, RMSD values were lower in the Pel3–Aesculin complex than in the unbound protein, reflecting enhanced structural stability, which may be directly attributable to better retention of internal hydrogen bonds. Similarly, the Rg analysis revealed that the complex exhibits a slightly larger but more stable radius of gyration, indicating a modest structural expansion without collapse—behavior that aligns with a preserved hydrogen bond network. SASA analysis also showed that, although the average surface exposure did not differ dramatically, the complex demonstrated superior surface stability, likely due to reduced internal fluctuations. Interestingly, both the solvent-accessible and internal hydrogen bond plots for the free Pel3 system exhibit greater fluctuation amplitudes, highlighting the inherently more flexible and potentially less stable nature of the unliganded protein. In contrast, the presence of Aesculin appears to mitigate these variations, resulting in a more stable and dynamically consistent system. From a drug design and structural biochemistry perspective, these findings are particularly valuable, as they suggest that the ligand not only binds to an active or allosteric site but also contributes to the global structural stabilization of the target protein.

### 3.4 RMSF and SASA analysis

**Fig. 4:**
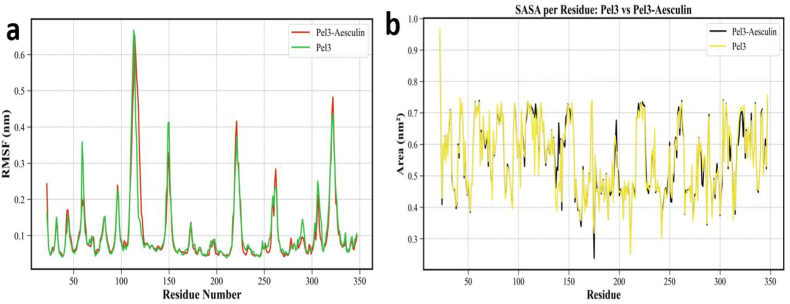
(A) Analysis of root mean square fluctuations (RMSF) and (B) solvent-accessible surface area (SASA) (B) for each residue in the unbound Pel3 protein and the Pel3-Aesculin complex.

Based on the data presented in Fig. 4, which includes two separate plots analyzing the RMSF and SASA of each residue in both the unbound Pel3 protein and the Pel3-Aesculin complex, a detailed assessment of the ligand's impact on the protein's dynamics and structural behavior can be conducted. The RMSF plot in Fig. 4a illustrates the spatial fluctuation magnitude of each residue throughout the MD simulation, serving as an indicator of local flexibility. In this plot, two curves corresponding to the unbound Pel3 protein (green) and the Pel3-Aesculin complex (red) are displayed simultaneously, enabling a direct comparison of the dynamic behavior between the two systems. The overall fluctuation patterns in both systems are largely similar, indicating that ligand binding preserves the general dynamic architecture of the protein. However, in many regions—particularly those proximal to the ligand-binding site—a relative decrease in RMSF values is observed in the complex compared to the unbound protein, reflecting a localized reduction in flexibility due to the stabilizing interactions between the ligand and the protein. The peak fluctuations in both systems occur approximately between residues 100 and 120, exceeding 0.65 nm, likely representing a flexible loop or terminal segment that remains highly mobile even after ligand binding. Notably, this peak is lower in the complex, clearly demonstrating the stabilizing effect of the ligand. Overall, the reduction in RMSF around the binding region can be interpreted as increased local rigidity resulting from spatial occupation by the ligand. According to the study by Lunin et al. (2015), the active site of Pel3 was identified, with Lys108 recognized as a critical catalytic residue. Interestingly, as shown in Fig. 4a, the highest fluctuation corresponds precisely to this region, particularly Lys108, suggesting that this residue is inherently flexible in the unbound state. The marked decrease in its mobility upon ligand binding further supports the role of Aesculin in stabilizing the active site conformation [34]. Conversely, a slight increase in RMSF is observed in some distal regions, which may arise from allosteric effects and structural rearrangements of the protein in response to ligand binding. As shown in Fig. 2B, the radius of gyration (Rg) of Pel3 in complex with Aesculin exhibits a slight increase beginning around 200 ns of the simulation, reaching an average of 1.97 ± 0.01 nm, compared to 1.95 ± 0.01 nm for the unbound protein. This subtle expansion suggests minor but notable conformational adjustments likely involving peripheral or flexible regions of the protein. Such local expansions do not compromise the global stability, as confirmed by a reduction in backbone RMSD values in the ligand-bound form (0.22 ± 0.03 nm) versus the free form (0.26 ± 0.05 nm). This observation is further supported by SASA analysis, where a marginal increase is observed in the complex (180.77 nm^2^) relative to the unbound state (180.34 nm^2^). Notably, this change in SASA is not global but rather localized, possibly reflecting the partial unfolding or surface rearrangement of specific loops or side chains upon ligand accommodation.

Fig. 4b displays the per-residue SASA, offering insight into the solvent exposure of individual residues and how surface accessibility is altered upon ligand interaction. In this graph, two curves corresponding to the unbound Pel3 (yellow) and the Pel3-Aesculin complex (black) are presented. Overall, SASA values for most residues are similar between the two systems, yet noticeable differences appear in certain regions. Around residues 50, 150, and 300, a clear decrease in SASA is observed in the complex, likely due to surface coverage by the ligand. This reduction in solvent-accessible area may suggest burial of side chains as a result of ligand interaction, serving as evidence for the physical proximity of the ligand to these regions. In other words, ligand binding results in regional rearrangements and variable surface accessibility, while maintaining the overall structural integrity of the protein. Based on the RMSF and SASA data, it can be concluded that the binding of Aesculin to Pel3 preserves the relative structural stability of the protein while reducing flexibility in specific regions. This localized rigidity at the binding interface may promote stronger interactions and enhance the retention of the ligand at the site. Nevertheless, the slight increase in flexibility observed in other regions implies retained dynamic behavior in the remaining protein structure, supporting its functional capabilities. These region-specific and targeted adjustments in response to ligand binding exemplify the phenomenon of allosteric regulation in biological systems. Additionally, the decrease in SASA for certain residues, particularly near the binding site, indicates partial burial of protein surface upon ligand complexation. This is typically associated with the formation of hydrophobic interactions and the local stabilization of protein structure. In contrast, the increase in SASA observed in other regions may reflect a structural relaxation aimed at compensating for geometric shifts induced by ligand binding. Although such rearrangements are minor and localized, they play a critical role in optimizing ligand-binding dynamics and preserving the biological activity of the protein. In summary, the combined results of Fig. 4A and B demonstrate that the Pel3-Aesculin complex possesses a stable yet moderately flexible structure, capable of maintaining global stability while exhibiting appropriate dynamic responsiveness to ligand binding. Such behavior is commonly observed in complexes involving shallow or intermediate binding pockets, where the ligand may engage in multiple transient binding modes and switch between them over time. Therefore, it can be concluded that Aesculin binding not only contributes to the stabilization of specific protein regions but also preserves the inherent flexibility necessary for the biological function of Pel3. The structural and dynamic properties observed for Pel3 in our simulations are consistent with prior crystallographic and functional studies. Gouet et al. (2008) revealed that PelI, a homologous PL-3 enzyme from Erwinia chrysanthemi, possesses a fibronectin type III N-terminal domain and a catalytic β-helix core, with ligand binding observed across a wide range of substrate subsites. The flexibility and functional separation between domains suggest dynamic rearrangements upon substrate or host interaction. Our RMSF analysis showing high fluctuations in loop regions supports this dynamic behavior [35]. Furthermore, Shevchik et al. (2021) demonstrated that specific flexible loop regions in Pel3 are essential for its secretion via the type II secretion system (T2SS), and their structural transitions likely contribute to substrate recognition [5]. In our study, these same flexible segments showed disorder-to-order reduction upon Aesculin binding, indicating that the ligand may play a stabilizing role not only in enzymatic inhibition but also in modulating secretion-relevant conformational states. These insights highlight the complex interplay between ligand binding, structural stability, and secretion competence in PL-3 enzymes, particularly Pel3 [5]. It is worth noting that while classical MD simulations were used in this study, recent advances in DFTB methods have demonstrated improved accuracy in modeling ligand–protein interactions by incorporating electronic effects, especially in large biomolecular systems [36,37]. Although DFTB calculations were beyond the scope of the current work, they represent a promising direction for future studies.

### 3.5 PCA and FEL analysis

**Fig. 5:**
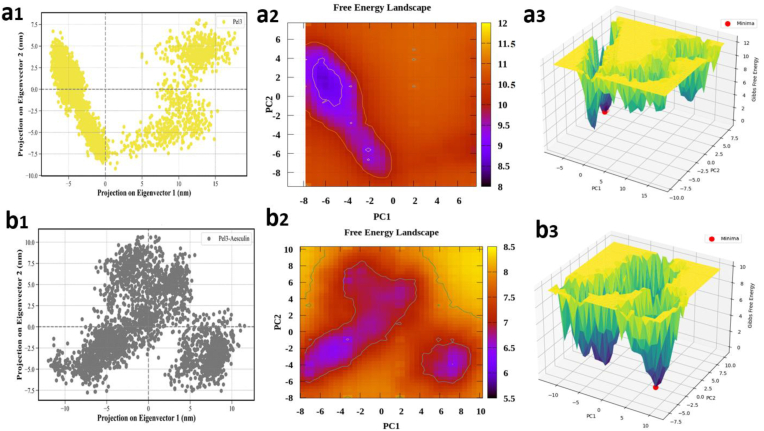
Principal component analysis (PCA) and free energy landscape (FEL) of the Pel3 protein and the Pel3–Aesculin complex. (a1) PCA projection of Pel3 in the absence of ligand, showing a compact and narrowly distributed conformational space along PC1 and PC2, suggesting a stable dominant motion. (a2) Two-dimensional FEL plot for free Pel3, revealing a single major energy minimum indicative of a predominant and stable conformational state. (a3) Three-dimensional FEL of free Pel3, confirming the presence of a deep, singular energy basin corresponding to a stable structural conformation. (b1) PCA projection of the Pel3–Aesculin complex, displaying broader dispersion and formation of multiple clusters, suggesting increased structural flexibility and diverse motions. (b2) Two-dimensional FEL of the complex, highlighting several distinct local minima, consistent with the emergence of multiple semi-stable conformational states. (b3) Three-dimensional FEL of the complex, illustrating multiple shallow energy valleys, further supporting the ligand-induced structural variability and dynamic adaptability.

In Fig. 5a1, principal component analysis (PCA) of the free form of the Pel3 protein reveals that the molecular dynamics data are tightly clustered with low dispersion along the PC1 and PC2 axes. This distribution pattern suggests that the dominant motions of the protein are restricted to a confined and relatively linear region of conformational space, indicative of structural stability and limited conformational diversity in the absence of a ligand. The average coordinates for the free form are reported as 4.87 on PC1 and –0.87 on PC2, and the low standard deviations further support the presence of a single dominant motion trajectory within the dynamic behavior of the protein. In contrast, Fig. 5b1, which illustrates PCA of the Pel3–Aesculin complex, shows a broader distribution of data points with the emergence of several distinct clusters. This expanded distribution along the principal axes indicates increased conformational variability and the presence of multiple structural states. The average coordinates for the complex are −0.62 on PC1 and 1.14 on PC2, with slightly higher standard deviations compared to the free form. Such a pattern may reflect greater structural flexibility upon ligand binding, potentially corresponding to an induced fit mechanism. Subsequently, the two-dimensional free energy landscape (FEL) depicted in Fig. 5a2 for the unbound protein reveals a single major energy minimum, indicating that in the absence of the ligand, the protein predominantly occupies one stable conformational state and rarely transitions to alternative states. Conversely, the FEL of the complex shown in Fig. 5B2 presents multiple discrete energy minima, suggesting that ligand binding induces the formation of several metastable conformations, potentially enabling conformational transitions within the binding site. While we do not have direct experimental validation, such conformational diversity may support functional adaptability, as observed in other lyase enzymes with flexible active sites. In the three-dimensional representations of the free energy landscape (Fig. 5a3 and 5b3), the contrast between the free and bound states becomes visually more apparent. Fig. 5a3 reveals a single, well-defined energy basin, in agreement with the two-dimensional FEL, again supporting the structural uniformity of the unbound protein. In Fig. 5b3, the presence of several energy basins with comparable depths indicates that the complex can adopt multiple stable conformations. This behavior reflects the enhanced dynamic potential of the ligand-bound form, which could have functional relevance in a biological context. The PCA and FEL results are consistent with other dynamic and structural stability indices, including RMSD, Rg, hydrogen bond count, and RMSF. Furthermore, the average number of hydrogen bonds formed between the protein and ligand during the simulation is higher in the complex, further supporting the structural stabilization conferred by ligand interaction. RMSF analysis demonstrates a reduction in flexibility in certain regions of the protein upon ligand binding, particularly near the binding site, which exhibits enhanced rigidity. This observation aligns with the PCA and FEL results, as ligand presence appears to stabilize specific conformational states and limit excessive fluctuations in functionally important regions. Additionally, while the overall SASA value does not markedly decrease in the complex compared to the free form, localized reductions in solvent exposure at the binding interface are observed, confirming effective ligand accommodation within the active site. Overall, the integration of all analyses indicates that binding of Aesculin to Pel3 not only enhances the global dynamic stability of the protein (evidenced by reduced RMSD and increased hydrogen bonding), but also introduces functionally relevant conformational flexibility, as reflected in the broader distribution in PCA and the presence of multiple energy minima in the FEL. Thus, ligand binding appears to establish a balance between overall structural stabilization and localized flexibility, which may contribute to improved biological function. These results suggest that while Aesculin binding increases the conformational landscape accessible to the protein—as evident from the broader PCA distribution and multiple FEL minima—it simultaneously stabilizes the global structure, as reflected in lower RMSD values and increased hydrogen bonding. This dynamic behavior may reflect a functionally favorable balance between structural integrity and localized flexibility, rather than instability.

### 3.6 Results of the dynamic behavior analysis of the Aesculin ligand in complex with the Pel3 enzyme using the RAMD method

To perform a detailed and comprehensive analysis of the ligand Aesculin dissociation behavior from the active site of the Pel3 enzyme, molecular dynamics simulations were conducted using the RAMD method on the Aesculin–Pel3 complex. A series of plots were generated using the tauRAMD-master tool, providing valuable insights into ligand residence times within the enzyme's active site, statistical data distributions, dissociation behavior, and evaluations of statistical tests such as the Kolmogorov–Smirnov (KS) test. The statistical parameters extracted from the RAMD simulations are summarized in Table 3. Subsequently, a comprehensive analysis of these plots is presented, offering both conceptual and statistical interpretations to help the reader gain a deeper understanding of the ligand's dynamic behavior in the enzymatic environment.

**Table 3:** Statistical parameters extracted from RAMD simulations.

Statistical parameter	Value
Total number of replications (Replicas)	120
Simulation time for each replication (ns)	50
Average residence time (ns)	0.015
Median residence time (ns)	0.01
Standard deviation of residence time (ns)	0.003
Maximum residence time (ns)	0.016
Minimum residence time (ns)	0.002
KS test statistic	0.17
KS test p-value	>0.05 (normality confirmed)

**Fig. 6:**
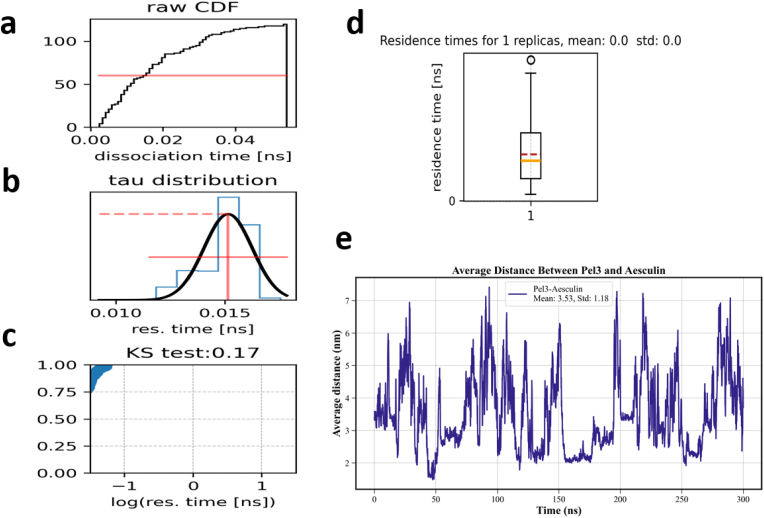
RAMD-based evaluation of ligand dissociation dynamics for the Pel3–Aesculin complex. (a) Cumulative distribution function (CDF), tau (residence time) histogram, and (b) Kolmogorov–Smirnov (KS) test plot illustrates the rapid unbinding of Aesculin from the Pel3 active site, with a median dissociation time of ∼0.02 ns and a mean residence time of ∼0.015 ns. (C) The residence time histogram shows a near-normal distribution, confirmed by a Gaussian fit and KS test (p = 0.17), supporting the statistical reliability of the data. (d) The boxplot summarizes residence time dispersion across a single simulation replica, highlighting the presence of outliers that may reflect alternative dissociation pathways. (e) Distance plot showing the temporal evolution of the ligand's center-of-mass distance from the protein's active site, providing direct evidence of dissociation events and their timing during simulation Overall, the results reveal fast ligand dissociation, limited residence time, and suggest weak binding affinity between Aesculin and Pel3 under the simulated conditions.

Fig. 6a displays three distinct but interrelated plots, arranged from top to bottom: the raw cumulative distribution function (CDF), the tau (residence time) distribution, and the KS test plot on a logarithmic scale. The second image shows a boxplot of residence times from a single replica simulation, providing complementary information on the statistical distribution of the data. The CDF represents the progressive accumulation of ligand dissociation events from the enzyme's active site. The x-axis denotes the dissociation time in nanoseconds (ns), while the y-axis reflects the cumulative count of dissociation events. A horizontal red line is drawn at y = 10, indicating that the intersection point of this line with the CDF curve corresponds to the time at which half of the simulation replicas have undergone ligand dissociation. This value is commonly used as a comparative metric for dissociation dynamics under varying conditions. Here, the dissociation time of the ligand is plotted against its residence time in nanoseconds. The median dissociation time (i.e., when 50 % of events have occurred) is approximately 0.02 ns, indicative of a rapid ligand unbinding from the active site. In Fig. 6b, labeled “tau distribution”, the residence time distribution of the ligand within the enzyme's active site is presented. The horizontal axis represents the residence time (in nanoseconds), while the vertical axis shows the frequency of occurrences. The blue curve corresponds to the histogram of empirical data derived from the simulation. Superimposed on this histogram is a black curve representing the Gaussian fit applied to the data. The good agreement between the fitted normal distribution and the empirical data suggests that the dataset approximately follows a normal distribution. This is important for validating the simulation results, as many statistical tests rely on the assumption of normality. A vertical red line on the same plot indicates the mean residence time. Based on its position, it can be inferred that the average residence time of the ligand in the active site is approximately 0.015 ns. Additionally, a horizontal dashed red line appears around y = 2, which may represent a normalized frequency or another measure of data concentration. The relatively low spread and tight clustering of the data indicate a stable ligand behavior within the enzymatic environment. Fig. 6c, titled “KS test: 0.17”, corresponds to the Kolmogorov–Smirnov (KS) test. The horizontal axis shows the logarithm of the residence time (log (res. time)), while the vertical axis represents the cumulative probability. This test compares the empirical distribution of the data with a reference distribution, typically normal or exponential. The p-value obtained from the test is 0.08, which exceeds the common significance level of 0.17. Thus, the null hypothesis of normality is not rejected, indicating that the data can be analyzed using classical statistical methods. This result confirms that the data are consistent with a reference distribution and are suitable for further statistical evaluation. Fig. 6d presents a boxplot of residence times, offering additional insights into data dispersion and distribution. The x-axis indicates the number of simulation replicas (in this case, only one was conducted), while the y-axis shows the residence time in nanoseconds. The box edges represent the first (Q1) and third quartiles (Q3), with the horizontal line inside the box denoting the median. The whiskers illustrate the range of non-outlier data points, and outliers are marked as individual circles. The reported mean and standard deviation are both zero, consistent with the appearance of the plot. However, this may be due to the limited number of replicas or insufficient sampling. It is important to emphasize that statistically valid conclusions require a larger number of independent simulations to ensure generalizability and robustness. From a biomolecular perspective, the residence time of a ligand in the enzyme's active site plays a critical role in determining the efficacy of an inhibitor or substrate. A short residence time indicates a high tendency of the ligand to dissociate from the active site, which may reflect weak binding, unstable interactions, or suboptimal spatial conformation. Conversely, longer residence times suggest potential binder with modest affinity interactions, such as stable hydrogen bonding, effective van der Waals contacts, or structural complementarity between the ligand and the active site. In this simulation, the mean residence time of 0.015 ns is considered relatively short, indicating rapid dissociation of the ligand from the enzyme. Several factors may contribute to this outcome: the spatial structure of Aesculin may not be well-aligned with the Pel3 enzyme's active site, the number or strength of hydrogen bonds and non-covalent interactions between ligand and enzyme may be low, simulation conditions such as a temperature of 300 K or the application of random acceleration in the RAMD method may facilitate faster ligand unbinding. Furthermore, the CDF plot confirms that the majority of dissociation events occurred within a very short timeframe (less than 0.04 ns), which is consistent with the other plots. Although the data are relatively concentrated, a number of outliers are also present, as highlighted by isolated circles in the boxplot. These outliers may reflect alternative dissociation pathways, suggesting the presence of multiple potential exit routes for the ligand—an observation frequently encountered in studies of protein dynamic mechanisms. In conclusion, the simulation results clearly indicate that the ligand Aesculin exhibits relatively low affinity and a short residence time in the active site of the Pel3 enzyme.

In a detailed analysis of the binding stability of the ligand Aesculin to the Pel3 protein, the average distance between these two molecules (Fig. 6e) serves as a key parameter for evaluating the quality and stability of their interaction. This distance is considered an indicator of spatial proximity and the ligand's tendency to remain within its binding site. Based on the distance–time plot, which illustrates the fluctuations in distance between the centers of mass of Aesculin and Pel3 over the 300 ns molecular dynamics simulation, the average distance was calculated to be approximately 3.53 nm with a standard deviation of 1.18 nm. The graph exhibits notable fluctuations throughout the simulation, indicating dynamic behavior of the ligand within the binding region. During certain intervals, the distance drops below 2 nm, suggesting deeper insertion into the binding pocket and potential binder with modest affinity interaction. Conversely, in other periods, the distance increases beyond 6 nm, even approaching 7 nm, which could imply transient instability, rotational movement, or spatial rearrangement of the ligand around the protein surface. When compared with other structural metrics, a more comprehensive understanding of system stability can be achieved. For example, this decrease in RMSD suggests that ligand binding contributes to enhanced structural stability of the protein by reducing fluctuations in the backbone. Such behavior implies that the presence of the ligand stabilizes the protein conformation and may prevent excessive flexibility. Furthermore, The Rg results showed that this slight increase was possible may result from structural interactions between the ligand and surface-exposed regions of the protein, potentially leading to partial opening of the structure or an increase in hydrodynamic size. Given the increased ligand–protein distances observed at certain time points in Fig. 6e, this outcome is justifiable and reflects the dynamic engagement of the ligand with various surface-exposed domains of the protein. Subsequently, an increase in SASA in the presence of the ligand generally indicates its interaction with the protein surface, which may expose structural elements or polar groups previously buried. These changes are consistent with the slight increase in Rg and the observed distance fluctuations between the ligand and the protein in Fig. 6e. Hydrogen bonding analysis (Fig. 2a and b, and Table 2) also reveals meaningful trends. Reducing the number of hydrogen bonds is likely due to the occupation of the binding site by Aesculin, which replaces some internal and external hydrogen bonding interactions with new ligand–protein contacts. This behavior aligns with the ligand–protein distance fluctuations, where closer interactions (distances below 3 nm) are expected to yield increased numbers of hydrogen bonds, as visually supported by Fig. 6e. From a dynamic perspective, the binding of Aesculin to Pel3 maintains overall structural stability while still permitting notable fluctuations in ligand position. This is evident from the time-dependent distance variations shown in Fig. 6e. Such fluctuations typically occur when the ligand occupies a surface-exposed or shallow binding site, which allows greater flexibility or implies the presence of multiple plausible binding modes. RAMD analysis, which aims to assess ligand–binding stability by applying random forces to facilitate unbinding, the time or energy required to dislodge the ligand is often compared to the average ligand–protein distance observed during classical simulations. In this study, with an average distance of 3.53 nm over 300 ns, it is expected that the energy required for Aesculin dissociation in RAMD would be relatively high, particularly during intervals where the distance drops below 2 nm. If RAMD simulations reveal that the ligand requires substantial force or extended time to dissociate, this would be well-aligned with the oscillatory but persistent interaction pattern presented in Fig. 6. In conclusion, analysis of RMSD, Rg, SASA, and hydrogen bonding patterns collectively supports the interpretation that the Pel3–Aesculin complex exhibits overall structural stability during the simulation. Although the ligand–protein distance shows dynamic fluctuations, these do not indicate complete dissociation or structural instability. Rather, they likely reflect Aesculin's occupancy of multiple binding modes, dynamically shifting between them—a behavior commonly observed in complexes involving surface or shallow binding sites.

### 3.7 MM-PBSA analysis

To complement the docking and molecular dynamics findings and provide a more quantitative thermodynamic perspective, MM-PBSA calculations were performed on the Pel3–Aesculin complex using snapshots extracted from the 300 ns MD trajectory. The total binding free energy (ΔG_bind) was estimated to be −2.92 ± 0.44 kcal/mol, indicating a moderate but favorable interaction between Aesculin and Pel3 (Table 4). van der Waals (−5.15 kcal/mol) and electrostatic (−2.78 kcal/mol) interactions contributed most significantly to the favorable binding, suggesting that Aesculin is primarily stabilized within the Pel3 active site via hydrophobic contacts and charge complementarity. In contrast, the polar solvation energy (+5.80 kcal/mol) exerted an opposing effect, consistent with desolvation penalties typically observed in aqueous systems. The overall gas-phase energy contribution (−7.94 kcal/mol) further reinforces the presence of strong intermolecular interactions. These MM-PBSA results are in close agreement with the docking-derived binding energy (∼–6.39 kcal/mol) and estimated inhibition constant (Ki ≈ 20.56 μM), supporting the classification of Aesculin as a moderate-affinity ligand. This level of interaction, while not indicative of tight binding, is consistent with a reversible and potentially biologically relevant inhibitory profile. Together with RMSD and hydrogen bonding analyses, the MM-PBSA data confirm that Aesculin binding enhances the structural stability of Pel3 without inducing significant destabilization or rigidification. Thus, Aesculin appears to confer a balance between stabilization and dynamic flexibility in the Pel3 structure, which may underlie its natural inhibitory potential.

**Table 4:** MM-PBSA binding free energy components for the Pel3–Aesculin complex over the 300 ns MD trajectory.

Energy Component	Average (kcal/mol)	SD (Standard Deviation)	SEM (Standard Error of Mean)
Bond	−0.00	0.00	0.00
Angle	0.00	0.22	0.00
Dihedral	−0.00	0.2	0.00
Urey-Bradley (UB)	0.00	0.03	0.00
Improper (IMP)	−0.00	0.02	0.00
CMAP	0.00	0.00	0.00
Van der Waals (VDWAALS)	−5.15	0.08	0.34
Electrostatic (EEL)	−2.78	0.40	0.50
1-4 Van der Waals	−0.00	0.00	0.00
1-4 Electrostatic	−0.00	0.00	0.00
Polar Solvation (EGB)	5.8	0.16	0.51
Nonpolar Solvation (ESURF)	−0.79	0.00	0.05
Gas Phase (GGAS)	−7.94	0.41	0.64
Solvation (GSOLV)	5.02	0.16	0.47
Total Binding Energy	−2.92	0.44	0.24

## 4 Conclusion

This study provides a comprehensive computational investigation into the interaction between Aesculin and the Pel3 enzyme using molecular docking, MD simulations, MM-PBSA binding free energy calculations, and RAMD-based residence time analysis. The findings collectively offer critical insights into the thermodynamics, structural dynamics, and inhibitory potential of Aesculin as a natural ligand. The 300 ns MD simulations revealed that Aesculin binding induces notable structural changes in the Pel3 protein. RMSD and Rg analyses indicate a reduction in structural fluctuations and suggest that Aesculin contributes to maintaining a more stable conformation of the enzyme. The slight increase in SASA and reduction in hydrogen bonds did not compromise structural integrity, further supporting a stabilizing interaction. These changes reflect a dynamic yet non-disruptive binding event, which may help preserve the enzyme's functional architecture under physiological conditions. MM-PBSA calculations estimated the total binding free energy (ΔG_bind) of the Pel3–Aesculin complex to be −2.92 ± 0.44 kcal/mol, indicating a moderate yet favorable binding interaction. van der Waals and electrostatic contributions were the primary stabilizing forces, while polar solvation energy introduced a desolvation penalty, a common feature in aqueous protein–ligand systems. The binding free energy profile is consistent with docking results (ΔG ≈ −6.39 kcal/mol) and an estimated inhibition constant (Ki ≈ 20.56 μM), supporting Aesculin's classification as a reversible and moderate-affinity inhibitor. To further assess the kinetic stability of the interaction, we expanded the RAMD simulations to 120 replicates, which yielded an average residence time of ∼0.015 ns and a median of ∼0.01 ns, based on a near-normal distribution. These updated kinetic parameters indicate that Aesculin exhibits a transient binding mode, with rapid dissociation from the Pel3 active site—likely due to suboptimal spatial complementarity. While this short residence time suggests a limited inhibitory duration, it also reflects the ligand's potential for rapid and reversible interactions, which may be beneficial in scenarios requiring transient inhibition or minimal off-target effects. Aesculin was selected as the ligand of interest due to its natural origin from the green husk of Juglans regia (walnut), where it is present in high abundance, as shown in our previous and ongoing phytochemical studies. Notably, Aesculin is known for its antibacterial and antifungal properties, making it a promising candidate for natural bioactive compound screening. Given that Pel3 is implicated in post-harvest spoilage of crops such as potatoes and vegetables, identifying natural inhibitors like Aesculin may offer a sustainable and safe approach to enhancing food preservation. In conclusion, although Aesculin does not exhibit tight binding or prolonged residence at the Pel3 active site, its moderate affinity, stabilizing structural effects, and natural antimicrobial origin position it as a viable scaffold for future optimization efforts. This study underscores the value of combining energetic, structural, and kinetic analyses to evaluate ligand potential and paves the way for the rational design of improved natural inhibitors targeting spoilage-related enzymes. Future work may explore structural analogs or derivatives of Aesculin with enhanced binding characteristics to expand its application in food safety and agricultural biotechnology.

## Author contributions

Ali Khakpour, Negar Ahmadi Shadmehri, Amir Sedaghati and Hassan Jamshidian Writing – original draft, Software, Methodology, contributed to conceptualization, methodology, validation. Shamim Ghiabi contributed to the methodology, interpretation of some results, writing. Seyedeh Atefeh Mirahmadi data curation, Writing, Software, Funding acquisition and Resources Ehsan Heidari-Soureshjani data curation, Validation, Software, Funding acquisition, Resources. Payam Baziyar contributed to methodology, supervision, data curation, Validation, formal analysis, writing—original draft, review and editing, Software, and Project administration.

## Generative AI statement

The author(s) declare that no Generative AI was used in the creation of this manuscript.

## Funding

The author(s) declare that no financial support was received for the research and/or publication of this article.

## Declaration of competing interest

The authors declare no conflict of interest.

## Data Availability

Data will be made available on request.

## References

[1] Amaya Guerrero A.P., Beltrán Pineda M.E., Alfonso Vargas N.C. (2021).

[2] Uluisik S., Seymour G.B. (2020). Pectate lyases: their role in plants and importance in fruit ripening. Food Chem..

[3] Bouley J., Condemine G., Shevchik V.E. (2001). The PDZ domain of OutC and the N-terminal region of OutD determine the secretion specificity of the type II out pathway of Erwinia chrysanthemi. J. Mol. Biol..

[4] Pineau C., Guschinskaya N., Robert X., Gouet P., Ballut L., Shevchik V.E. (2014). Substrate recognition by the bacterial type II secretion system: more than a simple interaction. Mol. Microbiol..

[5] Pineau C., Guschinskaya N., Gonçalves I.R., Ruaudel F., Robert X., Gouet P., Ballut L., Shevchik V.E. (2021). Structure–function analysis of pectate lyase Pel3 reveals essential facets of protein recognition by the bacterial type 2 secretion system. J. Biol. Chem..

[6] Chandler P.G., Buckle A.M. (2020). Development and differentiation in monobodies based on the fibronectin type 3 domain. Cells.

[7] Sidar A., Albuquerque E.D., Voshol G.P., Ram A.F., Vijgenboom E., Punt P.J. (2020). Carbohydrate binding modules: diversity of domain architecture in amylases and cellulases from filamentous microorganisms. Front. Bioeng. Biotechnol..

[8] Wattad C., Dinoor A., Prusky D. (1994). Purification of pectate lyase produced by Colletotrichum gloeosporioides and its inhibition by epicatechin: a possible factor involved in the resistance of unripe avocado fruits to anthracnose. MPMI-Mol. Plant Microbe Inter..

[9] Luo Y., Jian Y., Liu Y., Jiang S., Muhammad D., Wang W. (2022). Flavanols from nature: a phytochemistry and biological activity review. Molecules.

[10] Naghmachi M., Raissi A., Baziyar P., Homayoonfar F., Amirmahani F., Danaei M. (2022). Green synthesis of silver nanoparticles (AgNPs) by Pistacia terebinthus extract: comprehensive evaluation of antimicrobial, antioxidant and anticancer effects. Biochem. Biophys. Res. Commun..

[11] Khakpour A., Ghiabi S., Babaheydari A.K., Mirahmadi S.A., Baziyar P., Heidari-Soureshjani E., Horestani M.K. (2025). Discovering the therapeutic potential of naringenin in diabetes related to GLUT-4 and its regulatory factors: a computational approach. Chem. Phys. Impact.

[12] Abdullah Waheed Z., Seyedalipour B., Hosseinzadeh Colagar A., Baziyar P. (2024). Exploring the anti-amyloid potential of salvianolic acid A against the ALS-Associated mutant SOD1: insights from molecular docking and molecular dynamic simulations. Mol. Simul..

[13] Noorbakhsh Varnosfaderani S.M., Sadat Haeri M., Arian A.S., Yousefi Rad A., Yazdanpour M., Mojahedian F., Yaghoubzad-Maleki M., Zalpoor H., Baziyar P., Nabi-Afjadi M. (2025). Fighting against amyotrophic lateral sclerosis (ALS) with flavonoids: a computational approach to inhibit superoxide dismutase (SOD1) mutant aggregation. J. Biomol. Struct. Dyn..

[14] Qassim H.m., Seyedalipour B., Baziyar P., Ahamady-Asbchin S. (2023). Polyphenolic flavonoid compounds act as the inhibitory potential of aggregation process: implications for the prevention and therapeutics against FALS-associated D101G SOD1 mutant. Comput. Biol. Chem..

[15] Seyedi Asl F.S., Malverdi N., Ataei Kachouei F.S., Zarei F., Ghiabi S., Baziyar P., Nabi-Afjadi M. (2025). Inhibitory effect of Fisetin against the aggregation process of SOD1 E100K mutant: computer-based drug design as a potential therapeutic for ALS disease. Front. Chem..

[16] Zaji H.D., Seyedalipour B., Hanun H.M., Baziyar P., Hosseinkhani S., Akhlaghi M. (2023). Computational insight into in silico analysis and molecular dynamics simulation of the dimer interface residues of ALS-linked hSOD1 forms in apo/holo states: a combined experimental and bioinformatic perspective. 3 Biotech.

[17] Ebrahimi N., Far N.P., Fakhr S.S., Faghihkhorasani F., Miraghel S.A., Chaleshtori S.R., Rezaei-Tazangi F., Beiranvand S., Baziyar P., Manavi M.S. (2023). The endocannabinoid system, a new gatekeeper in the pharmacology of human hepatocellular carcinoma. Environ. Res..

[18] Asl F.S.S., Malverdi N., Mojahedian F., Baziyar P., Nabi-Afjadi M. (2025). Discovery of effective GSK-3β inhibitors as therapeutic potential against alzheimer's disease: a computational drug design insight. Int. J. Biol. Macromol..

[19] Sharbatdar Y., Mousavian R., Noorbakhsh Varnosfaderani S.M., Aziziyan F., Liaghat M., Baziyar P., Yousefi Rad A., Tavakol C., Moeini A.M., Nabi-Afjadi M. (2023). Diabetes as one of the long-term COVID-19 complications: from the potential reason of more diabetic patients' susceptibility to COVID-19 to the possible caution of future global diabetes tsunami. Inflammopharmacology.

[20] Li B., Li J., Akbari A., Baziyar P., Hu S. (2023). Evaluation of expression of cytochrome P450 aromatase and inflammatory, oxidative, and apoptotic markers in testicular tissue of obese rats (Pre) treated with garlic powder. Evid. base Compl. Alternative Med..

[21] Lamooki F.M., Alami F., Toupchi F.M., Jalali P., Banimahdidehkordi A., Baziyar P., Ghiabi S., Heidari-Soureshjani E., Mirahmadi S.A. (2025). Silymarin as a potent polyphenol against metabolic disease pathways: a computational insight and ADMET/MM-PBSA analysis. Results Chem..

[22] Bhat A.A., Shakeel A., Rafiq S., Farooq I., Malik A.Q., Alghuthami M.E., Alharthi S., Qanash H., Alharthy S.A. (2023). Juglans regia linn.: a natural repository of vital phytochemical and pharmacological compounds. Life.

[23] Kokh D.B., Wade R.C. (2021). G protein-coupled receptor–ligand dissociation rates and mechanisms from τRAMD simulations. J. Chem. Theor. Comput..

[24] D'Arrigo G., Kokh D.B., Nunes-Alves A., Wade R.C. (2024). Computational screening of the effects of mutations on protein-protein off-rates and dissociation mechanisms by τRAMD. Commun. Biol..

[25] Eberhardt J., Santos-Martins D., Tillack A.F., Forli S. (2021). AutoDock vina 1.2. 0: new docking methods, expanded force field, and python bindings. J. Chem. Inf. Model..

[26] Nabi-Afjadi M., Yaghoubzad Maleki M., Seyedi Asl F.S., Yazdanpour M., Ataei Kachouei F., Baziyar P. (2025). The effects of novel point mutations in the BRCA1 protein structure of breast cancer patients with breast or ovarian familial cancer history: an in-silico study. Euro. Phy. J. Plus.

[27] Ashkaran F., Seyedalipour B., Baziyar P., Hosseinkhani S. (2024). Mutation/metal deficiency in the" electrostatic loop" enhanced aggregation process in apo/holo SOD1 variants: implications for ALS diseases. BMC Chem..

[28] Faradonbeh S.M.H., Seyedalipour B., Behjou N.K., Rezaei K., Baziyar P., Hosseinkhani S. (2025). Structural insights into SOD1: from in silico and molecular dynamics to experimental analyses of ALS-associated E49K and R115G mutants. Front. Mol. Biosci..

[29] Farrokhzad R., Seyedalipour B., Baziyar P., Hosseinkhani S. (2025). Insight into factors influencing the aggregation process in wild‐type and P66R mutant SOD1: computational and spectroscopic approaches. Proteins: Struct., Funct., Bioinf..

[30] Flores-León C.D., Colorado-Pablo L.F., Santos-Contreras M.Á., Aguayo-Ortiz R. (2023). Determination of nucleoside DOT1L inhibitors' residence times by τRAMD simulations. Front. Drug Discov..

[31] Rios E.R.V., Rocha N.F.M., Venâncio E.T., Moura B.A., Feitosa M.L., Cerqueira G.S., Soares P.M.G., Woods D.J., de Sousa F.C.F., Leal L.K.A.M. (2010). Mechanisms involved in the gastroprotective activity of esculin on acute gastric lesions in mice. Chem. Biol. Interact..

[32] Tianzhu Z., Shumin W. (2015). Esculin inhibits the inflammation of LPS-induced acute lung injury in mice via regulation of TLR/NF-κB pathways. Inflammation.

[33] Khakpour A., Shadmehri N.A., Amrulloh H., Kioumarsi H. (2023). Antibacterial effect of Juglans regia, citrus sinensis, Vicia faba, and Urtica urens extracts under in vitro conditions. Bioactivities.

[34] Alahuhta M., Taylor L.E., Brunecky R., Sammond D.W., Michener W., Adams M.W., Himmel M.E., Bomble Y.J., Lunin V. (2015). The catalytic mechanism and unique low pH optimum of Caldicellulosiruptor bescii family 3 pectate lyase. Biol. Crystallograph..

[35] Creze C., Castang S., Derivery E., Haser R., Hugouvieux-Cotte-Pattat N., Shevchik V.E., Gouet P. (2008). The crystal structure of pectate lyase peli from soft rot pathogen Erwinia chrysanthemi in complex with its substrate. J. Biol. Chem..

[36] Allec S.I., Sun Y., Sun J., Chang C.-e.A., Wong B.M. (2019). Heterogeneous CPU+ GPU-enabled simulations for DFTB molecular dynamics of large chemical and biological systems. J. Chem. Theor. Comput..

[37] Sepay N., Chakrabarti S., Afzal M., Alarifi A., Mal D. (2022). Identification of 4-acrylamido-N-(pyridazin-3-yl) benzamide as anti-COVID-19 compound: a DFTB, molecular docking, and molecular dynamics study. RSC Adv..

